# 3D DenseNet Deep Learning Based Preoperative Computed Tomography for Detecting Myasthenia Gravis in Patients With Thymoma

**DOI:** 10.3389/fonc.2021.631964

**Published:** 2021-05-05

**Authors:** Zhenguo Liu, Ying Zhu, Yujie Yuan, Lei Yang, Kefeng Wang, Minghui Wang, Xiaoyu Yang, Xi Wu, Xi Tian, Rongguo Zhang, Bingqi Shen, Honghe Luo, Huiyu Feng, Shiting Feng, Zunfu Ke

**Affiliations:** ^1^ Department of Thoracic Surgery, The First Affiliated Hospital of Sun Yat-sen University, Guangzhou, China; ^2^ Department of Radiology, The First Affiliated Hospital of Sun Yat-sen University, Guangzhou, China; ^3^ Institution of Precision Medicine, The First Affiliated Hospital of Sun Yat-sen University, Guangzhou, China; ^4^ Center of Gastrointestinal Surgery, The First Affiliated Hospital of Sun Yat-sen University, Guangzhou, China; ^5^ Department of Thoracic Surgery, The Sun Yat-sen Memorial Hospital of Sun Yat-sen University, Guangzhou, China; ^6^ Advanced Institute, Infervision, Beijing, China; ^7^ Department of Neurology, The First Affiliated Hospital of Sun Yat-sen University, Guangzhou, China; ^8^ Department of Pathology, The First Affiliated Hospital of Sun Yat-sen University, Guangzhou, China

**Keywords:** thymoma, myasthenia gravis, deep learning—artificial neural network, computed tomography, imaging—computed tomography

## Abstract

**Background:**

Myasthenia gravis (MG) is the most common paraneoplastic syndromes of thymoma and closely related to thymus abnormalities. Timely detecting of the risk of MG would benefit clinical management and treatment decision for patients with thymoma. Herein, we developed a 3D DenseNet deep learning (DL) model based on preoperative computed tomography (CT) as a non-invasive method to detect MG in thymoma patients.

**Methods:**

A large cohort of 230 thymoma patients in a hospital affiliated with a medical school were enrolled. 182 thymoma patients (81 with MG, 101 without MG) were used for training and model building. 48 cases from another hospital were used for external validation. A 3D-DenseNet-DL model and five radiomic models were performed to detect MG in thymoma patients. A comprehensive analysis by integrating machine learning and semantic CT image features, named 3D-DenseNet-DL-based multi-model, was also performed to establish a more effective prediction model.

**Findings:**

By elaborately comparing the prediction efficacy, the 3D-DenseNet-DL effectively identified MG patients and was superior to other five radiomic models, with a mean area under ROC curve (AUC), accuracy, sensitivity, and specificity of 0.734, 0.724, 0.787, and 0.672, respectively. The effectiveness of the 3D-DenseNet-DL-based multi-model was further improved as evidenced by the following metrics: AUC 0.766, accuracy 0.790, sensitivity 0.739, and specificity 0.801. External verification results confirmed the feasibility of this DL-based multi-model with metrics: AUC 0.730, accuracy 0.732, sensitivity 0.700, and specificity 0.690, respectively.

**Interpretation:**

Our 3D-DenseNet-DL model can effectively detect MG in patients with thymoma based on preoperative CT imaging. This model may serve as a supplement to the conventional diagnostic criteria for identifying thymoma associated MG.

## Introduction

Thymoma is the most common neoplasm of the anterior mediastinum in adults and known for their frequent association with myasthenia gravis (MG) (1). MG, the most common syndrome of paraneoplastic syndromes (2), is an autoimmune disease, involving antibodies against the postsynaptic nicotinic acetylcholine receptors (AChRs) at neuromuscular junctions, resulting in variable weakness of the voluntary muscle (3). Patients with MG can experience severe cardiopulmonary complications (4, 5). One of the most severe complications of MG is myasthenic crisis after thymoma resection, which can rapidly worsen, leading to respiratory failure and even death (6–9). According to NCCN clinical guidelines for thymomas, all patients suspected of having thymomas (even those without symptoms) should be carefully evaluated for the presence of MG before surgical procedure in order to avoid respiratory failure during the operation (2, 10, 11). However, some MG symptoms are atypical or asymptomatic, leading to missed or delayed diagnosis of MG in patients experiencing mild weakness or in individuals with weakness restricted to only a few muscles (12). In addition, the current criteria for diagnosing MG (including immunological, electrophysiological, and pharmacological approaches) are usually complex and time-consuming (13). In the real world, a majority of thymoma patients did not receive careful evaluation of MG by a neurologist before surgery. Therefore, a simple, non-invasive and feasible screening method for detecting MG in thymoma patients prior to operation is necessary to ensure proper clinical management, especially for developing surgical strategies and reducing perioperative complications.

In recent years, deep learning (DL) and radiomics in the medical imaging field have been studied intensively to explore the potential of utilizing various medical images as diagnostic, predictive, or prognostic information of human diseases, including the possibility of identifying tumor pathological subtypes, tumor phenotypes, and the gene–protein signatures (14, 15). MG syndrome is closely related to the histopathological abnormalities of thymus, such as thymoma (16). Thymomas are usually stratified into six entities [types A, AB, B1, B2, B3, and TC (carcinoma)] on the basis of the morphology of epithelial cells and the lymphocyte-to-epithelial cell ratio (17). Thymoma associated MG is more common in type B (B_1_, B_2_, and B_3_) than type A and AB thymomas and absent in TC (16, 18). Recently, Xiaowei Han et al. reported that some CT imaging characteristics were significantly related to histological classification of thymoma and MG status (19). Therefore, it is possible to identify the presence of MG in thymoma patients using deep learning model or radiomics based on preoperative routine CT scan of thymoma.

Here, we designed this study to explore the effectiveness of 3D-DenseNet-DL model and five radiomics as predictive methods for MG using preoperative chest CT image. The final optimal model, named as 3D-DenseNet-DL based multi-model integrating with semantic CT image features, was ultimately established to detect MG in thymoma patients.

## Materials and Methods

### Patients

For this study, 182 patients diagnosed with thymoma who had undergone thymectomy at the First Affiliated Hospital of Sun Yat-sen University from Jan 1st, 2011 to Jun 31st, 2018 were included for analysis and model building (SYSUFH dataset, **Table 1**). Another 48 thymoma patients admitted to the Sun Yat-sen Memorial Hospital of Sun Yat-sen University from Jan 1st, 2017 to Mar 31st, 2019 were used as the external validation cohort (SYSUMH dataset, **Table 1**). All cases had undergone enhanced preoperative CT examination and had been clearly staged based on pathological examination and clinical manifestation. All patients in our study were evaluated by neurologists to determine the status of myasthenia gravis (MG) syndrome or other autoimmune diseases before operation and were followed up to 2 years after surgery. The diagnostic criteria of MG in this study includes: (1) typical clinical manifestations; (2) two or more of the following: (a) positive neostigmine test, (b) decline of >10% on electromyographic low-frequency repetitive nerve stimulation or increased jitter on single-fiber electromyography, (c) positive serum AChR-Ab or MuSK-Ab or LRP4-Ab. This project was approved by the Ethics Committee and Institutional Review Board of Sun Yat-sen University. Informed consent was waived due to the retrospective nature of this study.

**Table 1:** Baseline characteristic of the 230 patients with thymoma from two medical centers.

Variables	SYSUFH Dataset (n = 182)				SYSUMH Dataset (n = 48)			
	Number	without MG (n = 101)	with MG (n = 81)	P-value*	Number	without MG (n = 34)	with MG (n = 14)	P-value*
Sex				0.500				0.230
Male	115(63.2%)	66	49		27(56.3%)	21	6	
Female	67(36.8%)	35	32		21(43.7%)	13	8	
Age (year, mean ± SD)	NA	51.5 ± 13.1	47.5 ± 12.1	0.035^†^	NA	51.6 ± 13.8	50.4 ± 15.1	0.791^†^
WHO histologic classification				<0.001				0.227
A	22(12.1%)	19	3		8(16.7%)	7	1	
AB	37(20.3%)	22	15		13(27.1%)	11	2	
B1	21(11.5%)	15	6		5(10.4%)	4	1	
B2	72(39.6%)	26	46		19(39.6%)	10	9	
B3	21(11.5%)	10	11		3(6.25%)	2	1	
C	9(4.95%)	9	0		0	0	0	
Masaoka staging				0.006				0.151
I	84(46.2%)	43	41		41(85.4%)	28	13	
IIA	40(22.0%)	24	16		1(2.08%)	0	1	
IIB	16(8.79%)	5	11		0	0	0	
IIIA	20(10.9%)	13	7		5(10.4%)	5	0	
IIIB	13(7.14%)	7	6		1(2.08%)	1	0	
IV	9(4.94%)	9	0		0	0	0	
Smoking history				0.215				0.835
No	161(88.5%)	92	69		35(72.9%)	24	11	
Yes	21(11.5%)	9	12		13(27.1%)	10	3	
Surgical approach#				<0.001				0.051
Thymoma resection	31(17.0%)	30	1		4(8.33%)	4	0	
Thymectomy	30(16.5%)	29	1		30(62.5%)	23	7	
Extended thymectomy	111(61.0%)	34	77		14(29.2%)	7	7	

*Chi-square test or Fisher’s exact test; ^†^;Student’s t test; ^#^Some patients’ data were missing; NA, Not Applicable; SYSUFH, the First Affiliated Hospital of Sun Yat-sen University; SYSUMH, Sun Yat-sen Memorial Hospital of Sun Yat-sen University.

### CT Imaging Characteristics and Scan Protocol

Enhanced chest CT images were acquired within one week prior to operation. Imaging features were carefully evaluated through PACS reading workstation by two experienced radiologists specializing in chest CT imaging that were blinded to the MG statuses of the patients. CT Imaging characteristics that were evaluated included (**Table 2**): maximum diameter (3-D Maximum diameter); degree of enhancement (increment of enhanced CT value, HU); enhancement (homogeneous or heterogeneous); necrosis/cystic component (divided into 0–25%, 26–50%, 51–75%, 75–100% according to its volume percentage); shape (round or oval, lobulated, irregular); contours (smooth or irregular); presence of calcification, adjacent organ invasion, effusion (pleural/pericardial), and lymphadenopathy. All preoperative enhanced chest CT images were obtained with a 64-row multidetector CT scanner (Aquilion 64; Toshiba Medical, Tokyo, Japan). Scan parameters: x-ray tube voltage of 120 kVp; maximum of 500 mA with automatic tube current modulation. Axial thin-section CT images of the whole lung were reconstructed with a section thickness and spacing of 1.0 mm. Iopromide at 80–100 ml/per patient (300 mg I/m1, Schering Pharmaceutical Ltd) was injected at 3–4 ml/s flow rate and applied to contrast enhanced scanning protocol.

**Table 2:** Image characteristics of patients with thymoma.

Variables	Number	With MG		P-value
		No (n = 101)	Yes (n = 81)	
Maximum diameter^†^	NA	6.13 ± 2.93	4.91 ± 2.27	0.065
Degree of enhancement (HU)^†^	NA	32.56 ± 22.17	30.86 ± 20.06	0.972
Enhancement				0.074
Homogeneous	81(44.5%)	39	42	
Heterogeneous	101(55.5%)	62	39	
Necrosis/cystic component				0.029
0–25%	71(39.0%)	36	35	
26–50%	78(42.9%)	39	39	
51–75%	16(8.79%)	12	4	
75–100%	17(9.34%)	14	3	
Shape				0.027
Round or oval	91(50.0%)	50	41	
Lobulated	37(20.3%)	27	10	
Irregular	54(29.7%)	24	30	
Contours				0.030
Smooth	163(89.6%)	86	77	
Irregular	19(10.4%)	15	4	
Calcification				0.827
No	147(80.8%)	81	66	
Yes	35(19.2%)	20	15	
Adjacent organ invasion				<0.001
No	157(86.3%)	79	78	
Yes	25(13.7%)	22	3	
Effusion (Pleural/Pericardial)				0.028
No	169(92.9%)	90	79	
Yes	13(7.14%)	11	2	
Lymphadenopathy				0.030
No	166(91.2%)	88	78	
Yes	16(8.79%)	13	3	

^†^Data are mean ± standard deviation; NA, Not Applicable.

### Machine Learning

#### Datasets

Thymoma on CT images were segmented manually using the annotation tool “ITK-SNAP” (www.itksnap.org) (20). “ITK-SNAP”, as a free software, is widely used for medical image annotation and labeling. In this work, ITK-SNAP was applied for thymoma lesion segmentation. The output from ITK-SNAP is NIFTI files containing mask information of the thymoma for each sequence of CT images. We then used the mask information to extract the area of thymoma, namely the regions of interests (ROI) (**Figure S1**). For feature extraction in radiomic analysis, the segmented thymoma was used directly. For deep learning modeling, a further preprocessing step was designed to prepare the segmented data for the convolutional neural network.

#### Radiomic Analysis

##### Radiomic Analysis Procedure

Radiomic analysis involved several steps: feature extraction, feature selection and machine learning. First, feature extraction was performed to convert raw images to structural data with radiomic information that could be processed by machine learning algorithms. Then, several methods were applied to further select high-quality features based on variance or regression. Finally, the data with selected features are used as inputs for several mainstream machine learning algorithms to train and test the model.

##### Radiomic Features

The radiomic features were extracted using open source PyRadiomics software (http://pyradiomics.readthedocs.io) (21). The categories of features include: shape descriptors (2D and 3D), First Order Statistics, Gray Level Matrices (GLM) based: Gray Level Cooccurrence Matrix (GLCM), Gray Level Run Length Matrix (GLRLM), Gray Level Size Zone Matrix (GLSZM) and Gray Level Dependence Matrix (GMDM). These features were extracted not only from original images, but also from derived images filtered using Laplacians of Gaussians (LoG), Wavelet Decompositions, Square, Square Root, Logarithm and Exponential filters. In total, 1,390 radiomic features were extracted, covering the popular features used in research.

##### Radiomic Feature Selection

Feature selection was conducted to select a subset of features from all extracted features for use in model building. The aims of this step were to reduce the dimensions of features, simplify the model and enhance generalization by reducing overfitting. A multi-level selection approach was adopted, which involved three algorithms in the order of: variance threshold method, k-best method, and the least absolute shrinkage selection operator (LASSO). Variance based method was adopted at first to select features with variance larger than a threshold (threshold = 0.1 in this study, data were normalized to a range of −1 to 1). Then, top k (k = 300 in this paper) features were further selected based on top ANOVA F-value between feature and the label. Finally, LASSO with five-fold cross-validation was adopted to automatically select the more effective features (**Figure S2**).

##### Radiomic Model Building

The performance of radiomic analysis was evaluated using five popular machine learning algorithms: Random Forest, XGBoost, Multilayer Perceptron, Logistic Regression and Support Vector Machine.

#### Deep Learning

##### Data Preprocessing

For deep learning, images with fixed dimension 160 × 160 × 64 (pixels) were used as input of the model. The images were constructed with equal width and length of 160 pixels and channels of 64 pixels. The size of the input image was determined by statistical analysis of the region of all the thymomas in this dataset.

##### 3D-DenseNet

**Figure 1:**
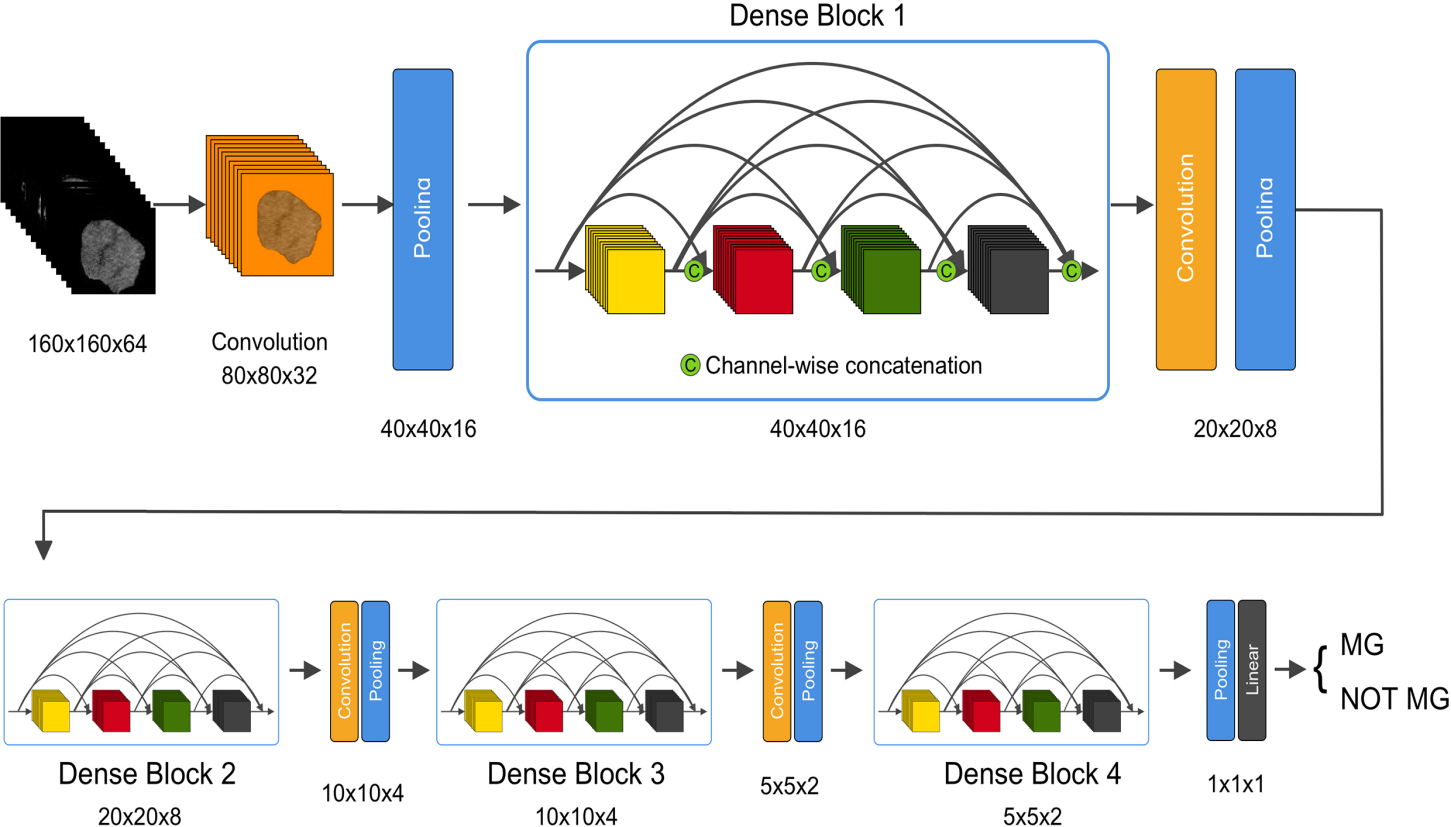
An illustration of the architecture of our 3D DenseNet deep learning model. Images with dimension 160 × 160 × 64 pixels are fed into the network, followed by multiple convolution and pooling operations, resulting in probability prediction for MG. In dense block, features with different levels are concatenated using skip connections. The dimension is halved after each transition layer.

DenseNet (22) is a type of convolutional neural network (CNN). DenseNet composes of four dense blocks, as shown in the schematic diagram. Dense connections between layers within dense blocks are present in DenseNet. We chose DenseNet as the base model in this study due to its various advantages. First, DenseNet can be used to reduce over-fitting. Second, DenseNet is computationally efficient as it requires less than half of the parameters of ResNet. Although DenseNet was first designed for two-dimensional images, our study targeted 3D CT sequences. As most medical images are three-dimensional, we designed a 3D-DenseNet where the kernel of each convolutional and pooling layer was modified to 3D versions. In the proposed 3D DenseNet model, rectified linear unit (ReLu) was used as activation function in each layer, and softmax function was applied in the last layer of our network to obtain the probability for each sample (**Figure 1** and **Table S1**). Batch normalization was applied before activation layer. The loss function of our model was due to binary cross-entropy, which was optimized using Adam with mini-batch size of 16.

##### Training Process Optimization

Two kinds of data augmentation were applied during the training stage of deep learning to avoid overfitting. First, random cropping (**Figure S3**) was implemented by randomly placing the segmented thymoma image in the fixed cube with shape. Second, a fixed window center (WC) and window width (WW) of 300 were applied for input images with original CT values. A random change was applied for the training data with WW value ranging from −10 to 10 and WC value from −5 to 5. Transfer learning was also applied to obtain benefit such as acceleration of the training stage from the pretrained model, which boosted the training speed significantly compared with the other initialization methods (such as Xavier).

##### Evaluation Metrics of Machine Learning

Five radiomic models (RF, XGBoost, SVM, MLP, LR) and one deep learning model (3D-DenseNet-DL model) were evaluated in the training and validation cohort using stratified five-fold cross-validation, and the parameter alpha was chosen with Mean Square Error (MSE) at minimum value. In this study, all the consecutively enrolled patients in SYSUFH dataset were randomly split into 80% for training and the remaining 20% for internal-validation. The metrics Area Under ROC Curve (AUC), ACC (accuracy), Sensitivity (SN) and Specificity (SP) were used to compare the performance of these models.

The deep learning model was implemented using MXNet (version 1.2.0, Apache Software Foundation, Forest Hill, MD, USA) library (22), and the model was trained using four NVIDIA GeForce GTX 1080 GPUs (NVIDIA, Beijing, China).

### Statistical Analyses

Statistical analyses were performed using SPSS 22.0 (IBM, USA). Variables were grouped based on the presence of MG. Categorical variables were compared using the Chisq test. Continuous variables were compared using the T-test or Mann–Whitney U test for variable with abnormal distribution. Multivariate logistic regression analysis was used to explore independent predictors of MG. Variables included in this analysis included age, gender, enhancement heterogeneity, necrosis/cystic component rate, contours, shape, adjacent organ invasion, pleural/pericardial effusion, and lymphadenopathy. p<0.05 was considered as statistically significant. The area under the ROC curve (AUC), accuracy, sensitivity, and specificity were measured in order to evaluate the accuracy of models.

## Results

### Clinical Characteristics of Patients

230 patients were included in this study; 182 (SYSUFH dataset) were used for training and model building, and an independent cohort of 48 cases (SYSUMH dataset) was used for external validation. The baseline characteristics of all patients from two medical centers were summarized in **Table 1**. In the SYSUFH cohort, a significant different ratio of histologic classification was found between the two groups (P < 0.001): MG patients with a lower A + AB ratio (22.2 *vs.* 40.6%) and higher B_1_ +B_2_ +B_3_ ratio (77.8 *vs.* 50.5%), compared to patients without MG. MG was not found in thymic carcinoma (TC), which is consistent with previous reports (18). In addition, MG patients showed significant association with younger age (47.5 ± 12.1 *vs.* 51.5 ± 13.1 years, P = 0.035) and relatively earlier thymoma Masaoka staging (P = 0.006). There were no significant differences between the two groups in terms of gender and smoking history.

### Associations Between Semantic CT Image Characteristics and Status of MG

Ten common variables were used to describe the CT imaging features of thymomas included in this study (**Table 2**). The statistical differences between two groups were found in necrosis/cystic component rate (P = 0.029), contours (smooth/irregular, P = 0.030), shape (P = 0.027), adjacent organ invasion (P < 0.001), pleural/pericardial effusion (P = 0.028), and lymphadenopathy (P = 0.030). In general, thymoma patients with MG tend to have less enhancement heterogeneity, less lobulated shape, and lower rate of adjacent organ invasion.

### Detection of MG by Radiomic Analysis and 3D DenseNet DL Model

For the radiomic analysis and deep learning (DL) analysis, a total of 1,390 radiomic features were extracted from the Routine contrast enhanced chest CT image data. After applying Variance Threshold, K-best and LASSO methods, the remaining features after application of each method were 499, 300, and 16, respectively. The 16 features finally selected were listed in **Table S2**. To decipher the relationship between features, correlation analysis using the Pearson method was applied, and a heatmap was constructed for visualization (**Figure S4**).

**Figure 2:**
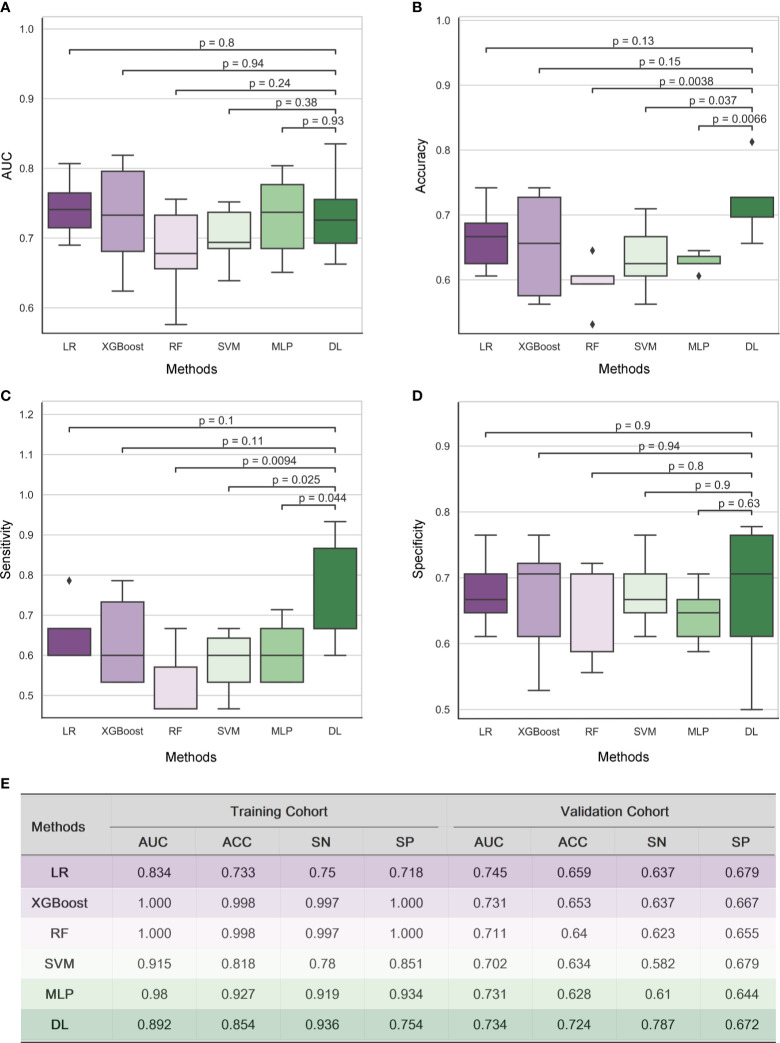
Results of Radiomic analysis and 3D DenseNet deep learning model for detecting MG in a cohort of 182 thymoma patients. The performance of five radiomic models and 3D-DenseNet-DL model was compared using Area Under ROC Curve (AUC) **(A)**, accuracy **(B)**, sensitivity **(C)**, and specificity **(D)**. 3D DenseNet deep learning model for detecting MG showed similar results in AUC and specificity, but relatively better results in accuracy and sensitivity compared to five radiomic analysis models **(E)**. “RF”, “LR”, and “DL” refer to “Random Forest”, “Logistic Regression”, and “Deep Learning” respectively; “AUC”, “ACC”, “SN”, and “SP” refer to the metrics Area Under ROC Curve, Accuracy, Sensitivity, and Specificity, respectively.

Five radiomic models (RF, XGBoost, SVM, MLP, LR) and 3D-DenseNet-DL model were established to detect the status of TAMG, and the values of each metric were shown in **Figure 2**. Compared with the other five radiomic models, the DL model showed the most favorable results with AUC 0.734, accuracy (ACC) 0.724, sensitivity (SN) 0.787 and specificity (SP) 0.672, respectively.

### Building of the 3D-DenseNet-DL Based Multi-Model for MG Detection

**Figure 3:**
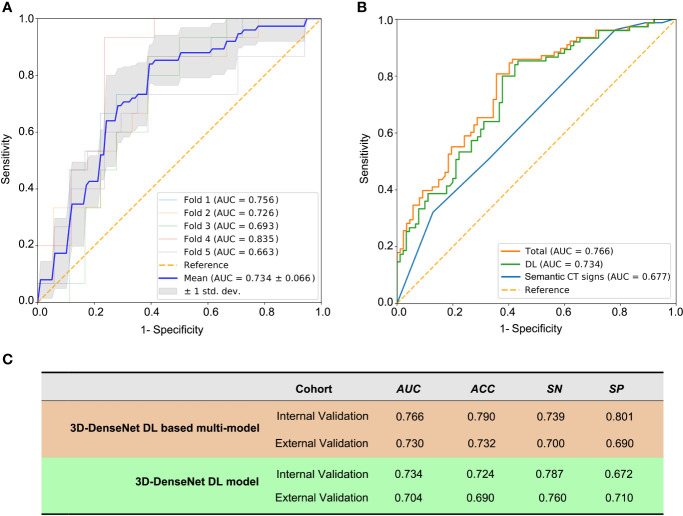
The prediction metrics of 3D-DenseNet-DL and DL based multi-model. The metrics Area Under ROC Curve (AUC), ACC (accuracy), SN (sensitivity), and SP (specificity) were used to compare the performance of these models. **(A)** The prediction metrics of the deep learning results from training and five-fold cross-validation, a mean AUC of 0.734 ± 0.066 was presented. **(B)** The comparison of three models of semantic CT signs model, 3D-DenseNet-DL model, and the comprehensive model (3D-DenseNet-DL based multi-model), with a mean AUC of 0.677, 0.734, and 0.766, respectively. **(C)** Values of 3D-DenseNet-DL model and 3D-DenseNet-DL based multi-model in external validation, with AUC 0.704, ACC 0.690, SN 0.760, and SP 0.710 for DL model, and AUC 0.730, ACC 0.732, SN 0.700, and SP 0.690 for our final 3D-DenseNet-DL based multi-model.

With the multivariable logistic regression analysis, only the shape of thymoma (P = 0.031), the invasion rate of adjacent organ (P = 0.001), and DL score (P< 0.001) qualified as independent predictable factors (**Table 3**). To optimize the effectiveness of TAMG-detecting model, we further built 3D-DenseNet-DL based multi-model (DL plus two semantic CT features). With ROC curve analysis, the AUC of DL model, semantic CT feature model (the shape and the invasion rate of adjacent organ), and 3D-DenseNet-DL based multi-model were 0.734, 0.677, and 0.766, respectively (**Figures 3A, B** **)**, suggesting that the 3D-DenseNet-DL based multi-model demonstrated better performance for detecting MG in thymoma patients.

**Table 3:** Significant correlations of MG with semantic CT imaging features and DL score using Logistic Regression Forward Stepwise (*Likelihood Ratio*) method.

Characteristic	P &	P #	OR # (95% CI)
Shape	0.031	0.032	1.59 (1.04–2.43)
Adjacent organ invasion	0.001	0.007	0.11 (0.02–0.54)
DL score	0.000	0.000	147.84 (9.15–1238.51)

OR, odd ratio; CI, confidence interval; DL, deep learning; #, The P value was calculated by multivariable logistic regression analysis adjusted for age and gender; &, Unadjusted P value; P < 0.05 was considered as statistically significant.

### The External Validation of 3D-DenseNet-DL Based Multi-Model

We further evaluated the 3D-DenseNet-DL model and the 3D-DenseNet-DL based multi-model in an external validation set composed of 48 thymoma patients from another medical center (SYSUMH). The results showed a comparable agreement in both datasets for the detection of TAMG, with an AUC of 0.730, ACC of 0.732, SN of 0.700, and SP of 0.690 for 3D-DenseNet-DL based multi-model; and AUC of 0.704, ACC of 0.690, SN of 0.760, and SP of 0.710 for 3D-DenseNet-DL model (**Figure 3C**). This favorable result further confirmed the reliability and efficacy of our 3D-DenseNet-DL based multi-model in screening TAMG in patients with thymoma.

## Discussion

In this study, we proposed and validated a non-invasive method based on preoperative routine CT imaging of thymoma, referred to as “3D DenseNet deep learning (DL) based multi-model”, to detect MG before operation. With this model, we successfully filtered out most of MG patients in the internal validation set (AUC of 0.766), and further verified its reliability and efficacy in an external validation set (AUC of 0.730). We also established five radiomic models (RF, XGBoost, SVM, MLP, LR) to detect MG and compared the effectiveness in detecting disease with the DL model. Our results suggest our 3D-DenseNet-DL based multi-model is an effective and non-invasive method for detecting MG in patients with thymoma. To our knowledge, this is the first study about the diagnosis of MG in thymoma patients by using machine learning based on CT imaging data.

Currently, a large number of diagnostic tests are available for MG diagnosis, including clinical, electrophysiological, and laboratory antibody tests (23). However, results from these tests can be negative in some patients, and diagnosing pure ocular MG can be a challenge (23). The anti-AChR antibody assay by radioimmunoassay kits is considered as the most reliable approach to diagnose MG (24, 25). However, these kits are expensive and not routinely available for most diagnostic laboratories. In China, commercial enzyme-linked immunosorbent assay (ELISA) kits are more widely used for anti-AChR test, which are inferior to radioimmunoassay in terms of both sensitivity and specificity (26). Therefore, although AChR antibody is found in nearly all of thymoma associated MG patients, the false positive rate was also high (16). Repetitive nerve stimulation (RNS) (27) and single-fiber electromyography (SFEMG) (28) are also used in electrophysiological confirmation for MG. However, SFEMG may not provide confirmation of the presence of MG unless weak muscles are tested, and the reliability of results is highly dependent on the experience of the technician (13). More importantly, as a relatively rare autoimmune disease, the diagnosis system of MG is mainly concentrated in large hospitals or medical centers, and a large number of patients with thymoma cannot be effectively screened for MG before surgery. Considering the limitations or unavailability of these classical diagnostic methods, a simple, easily available method with good efficacy is important in clinical practice for the preoperative detecting of MG in thymoma patients. CT examination, as a routine preoperative examination for patients with thymoma, is very popular. Therefore, our MG detecting DL model based on preoperative CT has great application prospects and clinical significance.

Nowadays, an increasing number of studies are performed to evaluate the potential relationship between CT imaging and biological features of solid tumors (29), such as glioblastoma (30), rectal and lung adenocarcimoma (31, 32). As the most common primary neoplasms of the mediastinum, the prediction of thymoma histology and stage by radiographic criteria has been mentioned in several previous reports. CT findings, such as smooth contours (33), calcification (33, 34), heterogeneous attenuation (34, 35), were interpreted as being of value in differentiating the various histologic subtypes of thymomas. Recently, Angelo lannarelli and colleagues (36) found a relationship between radiomic parameters, histology, and grading of thymic tumors. More importantly, their study also demonstrated that MG syndrome was significantly associated with some parameters in quantitative texture analysis (QTA) (36). Although their study only included 16 patients (seven patients with MG), it represented an incentive for further evaluation of the value of radiographic analysis in detection of MG syndrome in thymoma patients. Based on these findings, we therefore proposed a machine learning model based on preoperative CT imaging for screening MG in large cohort of thymoma patients and achieved the expected results. Moreover, our results further confirmed the superior reliability and efficacy of this developed 3D-DenseNet-DL model compared to the other five radiomic-based methods. These results also highlight the importance of radiographic analysis as diagnostic tools from the accurate characterization of the lesion itself to the detection of the paraneoplastic syndromes, which is a great stride in the application of AI in the medical field.

However, despite its satisfactory outcomes, this study has some limitations. First, given the retrospective nature of this analysis, a selection bias was unavoidable. Second, patients were not stratified into more detailed clinical status categories due to limited sample size. Third, the status of serum AChR binding antibodies was important for thymoma associated MG diagnosis, but the absence of such information in certain cases restrained further analysis. In addition, our deep learning model was built and validated only based on pathologically diagnosed thymoma, which limits the application scope of this model to some extent. Therefore, a perspective, multi-center clinical trial with larger cohort would be indispensable to further confirm and optimize the screening model for MG patients.

In conclusion, with a large sample data for modeling and an independent cohort for external validation, we firstly developed a 3D-DenseNet-DL based multi-model for MG screening in thymoma patients based on preoperative CT imaging and achieved favorable results.

## Data Availability Statement

The raw data supporting the conclusions of this article will be made available by the authors, without undue reservation.

## Author Contributions

Study concept and design, literature search, and writing: ZL, YZ, HF, HL, SF, and ZK. Data collection/interpretation: YZ, HF, YY, LY, BS, HL, KW, and MW. Data analysis: ZL, YZ, XY, XW, XT, SF, HL, and RZ. Approval of final version of submitted manuscript: all authors. Manuscript editing: all authors. All authors contributed to the article and approved the submitted version.

## Funding

The work was supported by grants from China National Natural Sciences Foundation (No.82001331) and Natural Sciences Foundation of Guangdong Province (No.2018A0303130250).

## Conflict of Interest

Authors XT and RZ were employed by Advanced Institute, Infervision, Beijing.

The remaining authors declare that the research was conducted in the absence of any commercial or financial relationships that could be construed as a potential conflict of interest.

## Abbreviations

ACC, accuracy; AChR, acetylcholine receptors; AI, artificial intelligence; AUC, area under ROC curve; CNN, convolutional neural network; CT, computed tomography; DenseNet, densely connected convolutional networks; DL, deep learning; MG, masthenia gravis; PNS, paraneoplastic syndromes; ROI, regions of interests; SN, sensitivity; SP, specificity; TC, thymic carcinoma; RF, random forest; LR, logistic regression.
