# Supplementary material for: 3D DenseNet Deep Learning Based Preoperative Computed Tomography for Detecting Myasthenia Gravis in Patients With Thymoma
Source: Front Oncol. 2021 May 5;11:631964. doi: 10.3389/fonc.2021.631964 (PMC8132943; doi:10.3389/fonc.2021.631964)
Supplement: Supplementary file 1 [file DataSheet_1.pdf]

Figure supplementary

Figure S1

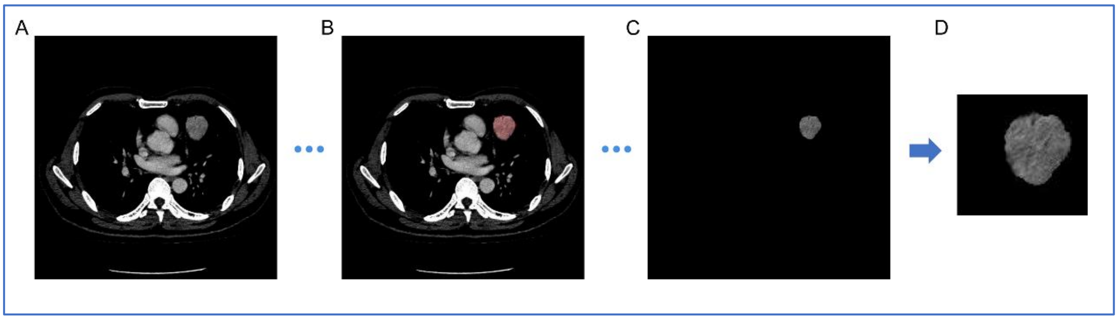

Figure S1. Datasets image example and image processing. A, shows one original slice of CT series including the thymoma region; B, illustrates the annotated image using the related image mask; C, is the segmented thymoma region; D, is the thymoma region that excludes the irrelevant surroundings, used for radiomics feature extraction and deep learning model.

Figure S2

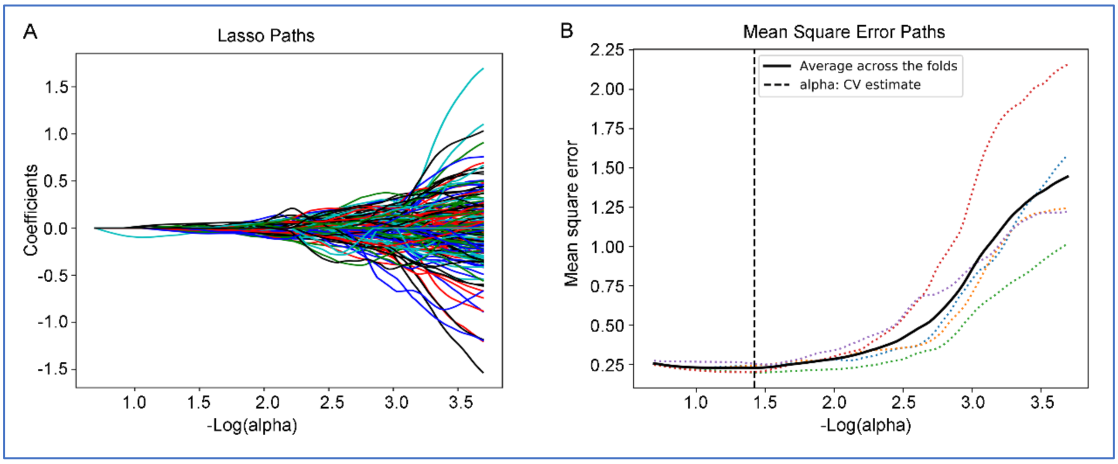

Figure S2. The parameter tuning in LASSO method. Figure A shows the relevance affected by different regularization coefficient alpha, and features with non-zero coefficients can be identified as relevant features; (B) By using five-fold cross validation in LASSO, the coefficients alpha can be chosen when Mean Square Error (MSE) has minimum value. Then, relevant features can be selected based on alpha.

Figure S3

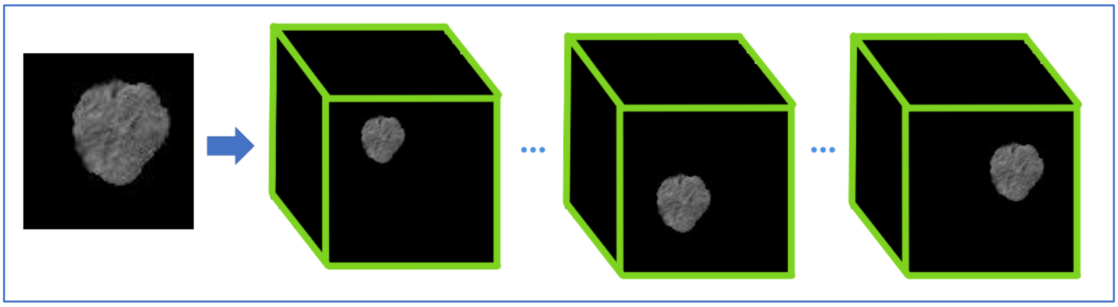

Figure S3. The data augmentation using random cropping. The left is the original segmented thymoma ROI, and the right is random generated training samples which are illustrated as cubes. During training, the thymoma ROI can be placed in any position within the cube to increase data variability.

Figure S4

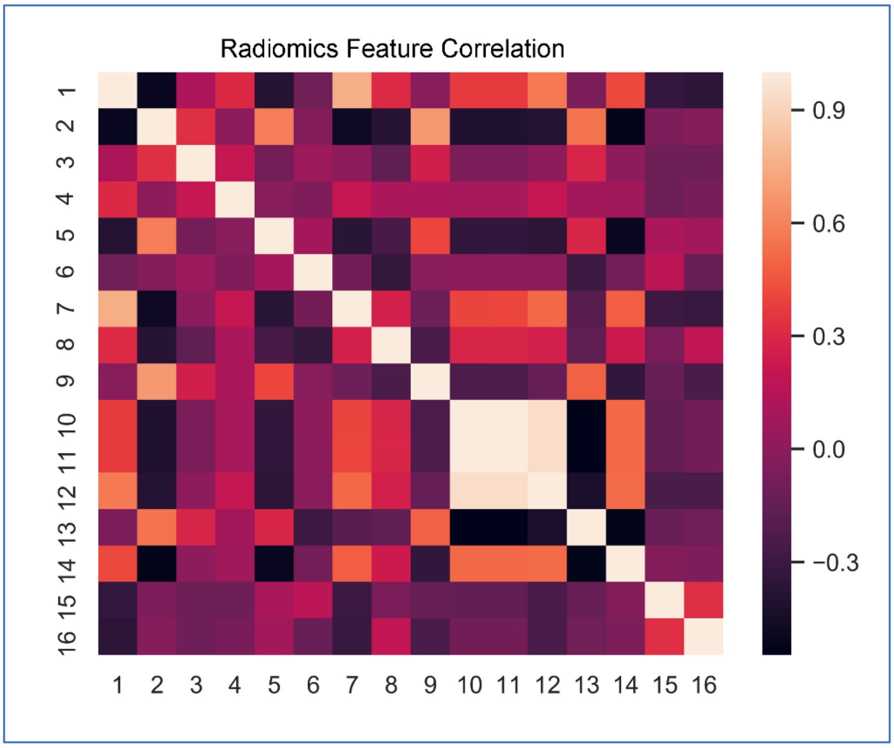

Figure S4. The heatmap of radiomics feature correlation analysis. The heatmap shows the correlation coefficient ranging from -1 to 1 for the extracted 16 features. It can be seen that the majority of these features have lower correlation coefficients, meaning that redundant features with higher coefficients have been removed from the original extracted features. The correlation between features was analyzed using Pearson method.
