# Supplementary material for: 3D DenseNet Deep Learning Based Preoperative Computed Tomography for Detecting Myasthenia Gravis in Patients With Thymoma
Source: Front Oncol. 2021 May 5;11:631964. doi: 10.3389/fonc.2021.631964 (PMC8132943; doi:10.3389/fonc.2021.631964)
Supplement: Supplementary file 2 [file DataSheet_2.pdf]

Table supplementary

Table S1: The structure of the 3D-DenseNet.

Layers	Output Size (pixels)	Operation
Conv	80 × 80 × 32	7 × 7 × 7 Conv, Stride 2
Pooling	40 × 40 × 16	3 × 3 × 3 Max Pool, Stride 2
Dense Block 1	40 × 40 × 16	6 × (3 × 3 × 3 BottleNeck-Conv)
TL-Conv 1	40 × 40 × 16	1 × 1 × 1 Conv
TL-Pooling 1	20 × 20 × 8	2 × 2 × 2 Average Pool, Stride 2
Dense Block 2	20 × 20 × 8	12 × (3 × 3 × 3 BottleNeck-Conv)
TL-Conv 2	20 × 20 × 8	1 × 1 × 1 Conv
TL-Pooling 2	10 × 10 × 4	2 × 2 × 2 Average Pool, Stride 2
Dense Block 3	10 × 10 × 4	24 × (3 × 3 × 3 BottleNeck-Conv)
		36 × (3 × 3 × 3 BottleNeck-Conv)
		32 × (3 × 3 × 3 BottleNeck-Conv)
		48 × (3 × 3 × 3 BottleNeck-Conv)
TL-Conv 3	10 × 10 × 4	2 × 2 × 2 Average Pool, Stride 2
TL-Pooling 3	5 × 5 × 2	
Dense Block 4	5 × 5 × 2	16 × (3 × 3 × 3 BottleNeck-Conv)
		24 × (3 × 3 × 3 BottleNeck-Conv)
		32 × (3 × 3 × 3 BottleNeck-Conv)
		32 × (3 × 3 × 3 BottleNeck-Conv)
Pooling	1 × 1 × 1	3 × 3 × 4 Global Average Pooling
FC Layer		Fully Connected Layer, Softmax

Note: Conv, convolutional operation, by default including batch normalization and activation layer; BottleNeck-Conv, layer that includes 1×1×1 convolution before regular convolutions for parameters reduction; TL-Conv or TL-Pooling, transition layers to connect dense blocks.

Table S2. 16 selected radiomic features in the final analysis.

Feature Name	Descriptions
Sphericity	Roundness of the shape of ROI.
LeastAxisLength	The smallest axis length of the ROI-enclosing ellipsoid.
Flatness	The relationship between the largest and smallest principal component.
square_glcm_Correlation	Linear dependency of gray level values to voxels.
log-sigma-3-0-mm-3D_firstorder_Maximum	The maximum gray level intensity within the ROI.
wavelet-LHL_glcm_Correlation	
wavelet-LHH_glszm_SmallAreaLowGrayLevelEmphasis	The proportion in the image of the joint distribution of smaller size zones with lower gray-level values.
wavelet-LLH_firstorder_Skewness	The asymmetry of the distribution of values about the Mean value.
wavelet-HLH_glszm_LargeAreaHighGrayLevelEmphasis	the proportion in the image of the joint distribution of larger size zones with higher gray-level values.
wavelet-HHH_gldm_LowGrayLevelEmphasis	The distribution of low gray-level values, with a higher value indicating a greater concentration of low gray-level values in the image.
wavelet-HHH_glrlm_LowGrayLevelRunEmphasis	
wavelet-HHH_glrlm_LongRunLowGrayLevelEmphasis	Joint distribution of long run lengths with lower gray-level values.
wavelet-HHH_glszm_SmallAreaEmphasis	Distribution of small size zones, with a greater value indicative of more smaller size zones and more fine textures.
wavelet-HHL_glrlm_LowGrayLevelRunEmphasis	
wavelet-LLL_glcm_Imc2	The correlation between several probability distributions.
wavelet-LLL_gldm_DependenceNonUniformityNormalized	The similarity of gray-level intensity values in the image

Note: prefix of “square”, “log” and “wavelet-” represent for different image filters.

Table S3. The prediction metrics of the deep learning results from training and five-fold cross-validation.

	**AUC**	**Accuracy**	**Precision**	**Sensitivity**	**Specificity**
Training					
Fold 0	0.879	0.812	0.750	0.919	0.712
Fold 1	0.886	0.867	0.795	0.984	0.754
Fold 2	0.989	0.930	0.877	0.990	0.859
Fold 3	0.896	0.844	0.772	0.968	0.723
Fold 4	0.811	0.766	0.739	0.810	0.723
mean	0.892	0.844	0.787	0.936	0.754
Validation					
Fold 0	0.756	0.727	0.714	0.667	0.778
Fold 1	0.726	0.727	0.650	0.867	0.611
Fold 2	0.693	0.697	0.609	0.933	0.500
Fold 3	0.835	0.812	0.765	0.867	0.765
Fold 4	0.663	0.656	0.643	0.600	0.706
mean	0.734	0.724	0.676	0.787	0.672
